# Deep learning in precision medicine and focus on glioma

**DOI:** 10.1002/btm2.10553

**Published:** 2023-05-31

**Authors:** Yihao Liu, Minghua Wu

**Affiliations:** ^1^ Hunan Key Laboratory of Cancer Metabolism, Hunan Cancer Hospital and the Affiliated Cancer Hospital of Xiangya School of Medicine Central South University Changsha Hunan China; ^2^ NHC Key Laboratory of Carcinogenesis, Xiangya Hospital Central South University Changsha Hunan China; ^3^ Key Laboratory of Carcinogenesis and Cancer Invasion of the Chinese Ministry of Education, Cancer Research Institute Central South University Changsha Hunan China

**Keywords:** artificial intelligence, deep learning, glioma, machine learning, precision medicine

## Abstract

Deep learning (DL) has been successfully applied to different fields for a range of tasks. In medicine, DL methods have been also used to improve the efficiency of disease diagnosis. In this review, we first summarize the history of the development of artificial intelligence models, demonstrate the features of the subtypes of machine learning and different DL networks, and then explore their application in the different fields of precision medicine, such as cardiology, gastroenterology, ophthalmology, dermatology, and oncology. By digging more information and extracting multilevel features from medical data, we found that DL helps doctors assess diseases automatically and monitor patients' physical health. In gliomas, research regarding application prospect of DL was mainly shown through magnetic resonance imaging and then by pathological slides. However, multi‐omics data, such as whole exome sequence, RNA sequence, proteomics, and epigenomics, have not been covered thus far. In general, the quality and quantity of DL datasets still need further improvements, and more fruitful multi‐omics characteristics will bring more comprehensive and accurate diagnosis in precision medicine and glioma.

**Abbreviations**
Adaline: adaptive linear element
AI: artificial intelligence
AUPRC: the area under the precision‐recall curve
AUROC/AUC: the area under the receiver operating characteristic curve
BP: back propagation
BraTS: brain tumor segmentation challenge
CART: classification and regression tree
CNN: convolutional neural network
DBN: Deep Belief Net
DenseNet: Dense Convolutional Network
DL: deep learning
DNNs: deep neural networks
DSC: dice similarity coefficient
ECG: electrocardiogram
ECOC‐SVM: error‐correcting output code support vector machine
eICU: eICU Collaborative Research Database
Faster RCNN: faster region‐based convolutional network
FCN: fully convolutional network
GAN: generative adversarial network
GBM: gradient boosting machine
HMDB: Human Metabolome Database
IDH: isocitrate dehydrogenase
IoU: intersection over union
ISIC: International Skin Imaging Collaboration
ISOMAP: isometric feature mapping
KEGG: Kyoto Encyclopedia of Genes and Genomes
KNN: K‐nearest neighbor
KPCA: Kernel Principal Component Analysis
LASSO: Least Absolute Shrinkage and Selection Operator
LLE: locally linear embedding
LSTM: long short‐term memory
MGMT: O^6^‐methylguanine‐DNA methyltransferase
MIMIC‐III: Medical Information Mart for Intensive Care III
ML: machine learning
MLP: multi‐layer perceptron
MRI: magnetic resonance imaging
MURA: Stanford's Musculoskeletal Radiographs
OCT: optical coherence tomography imaging
PDB: Protein Data Bank
PSNR: peak signal‐to‐noise ratio
ResNet: residual neural network
RNNs: recurrent neural networks
SENet: Squeeze‐and‐Excitation Network
SVM: support vector machine
t‐SNE: t‐distributed stochastic neighbor embedding
TCGA: The Cancer Genome Atlas
TCIA: The Cancer Imaging Archive
UK Biobank: UK Biobank Imaging Study
VAE: variational autoencoder
ViT: vision transformer
WHO: World Health Organization
WSIs: whole slide images
XGBoost: extreme gradient boosting

## 1 INTRODUCTION

Medicine and clinical work generate a massive amount of data from various sources, such as multiple biosensors, high‐resolution medical imaging, electronic medical records, and genome sequencing. The sheer size of these medical data makes it impossible for doctors to manually process patient data and assess detailed biological information for patients, making it an obstacle to precision and personalized medicine. Thus, the assistance of computers and digital tools has become increasingly vital. Deep learning (DL), for example, has been found to be an efficient tool for handling such conditions.

Precision medicine is the tailoring of healthcare and clinical decisions to patients based on their intrinsic biological information and clinical signs and symptoms. In 2011, the National Academy of Sciences proposed that genomic achievements promote the integration of biomedical informatics and clinical informatics, thus kickstarting the era of precision medicine.^1^ Precision medicine and personalized medicine aim to better diagnose and treat specific patients. Precision medicine serves the need for new classifications of diseases at the molecular level by integrating biomedical research and clinical medical information. These new disease subtypes provide a more accurate diagnosis and treatment plan. Precision medicine and personalized medicine complement each other and have become the development trend of modern medicine.

**FIGURE 1:**
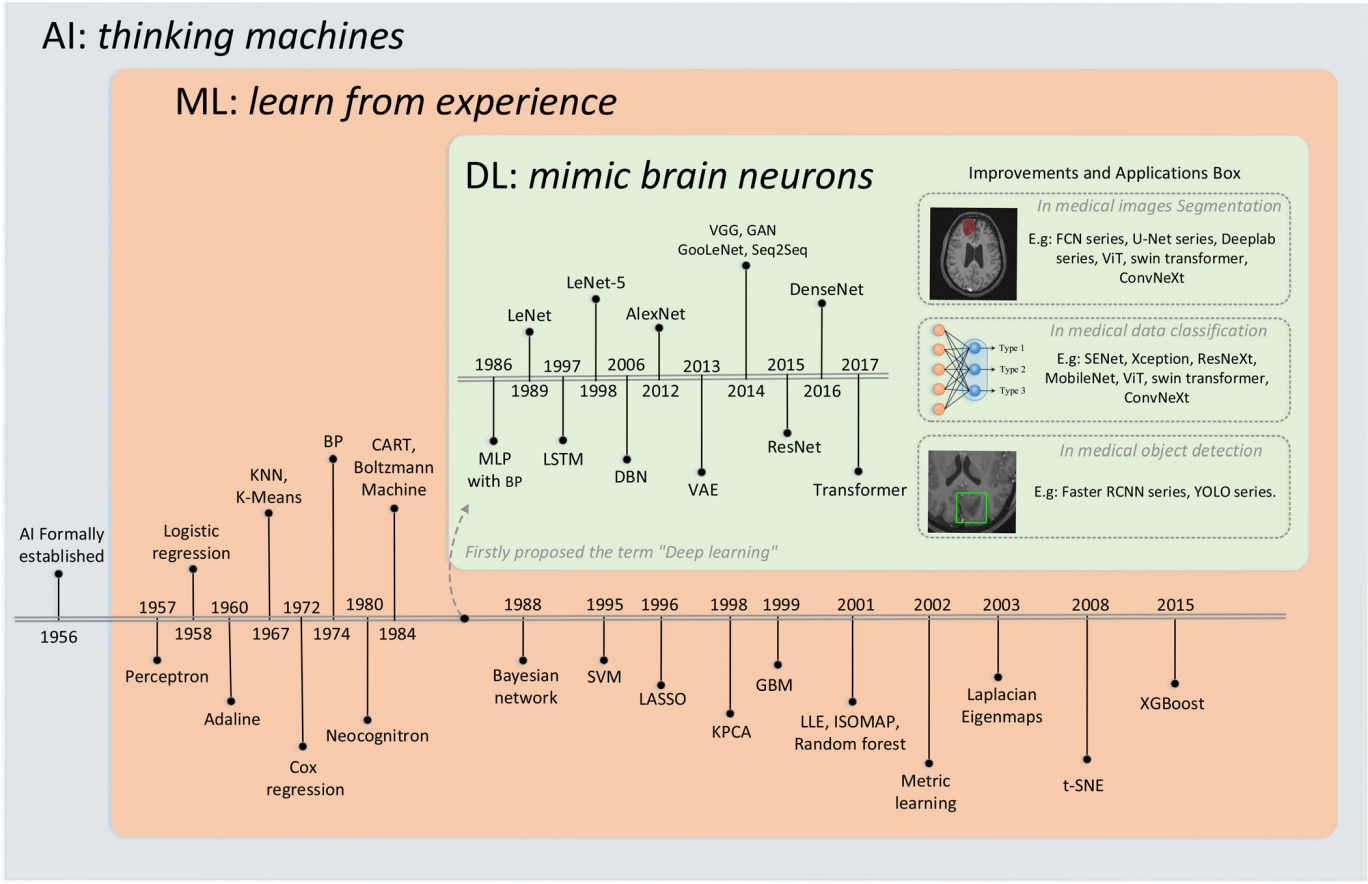
Developments history of AI models. Model structures improved from simple to integrated and complex (from decision tree to boosting series algorithm, from MLP to transformer), feature extraction/engineering improved from manual to automated (from traditional machine learning to deep learning), and application fields from limited to wide. Various applications emerged with the improvements of AI and a brief list of their corresponding networks are showed in the right side of figure. Full names of the abbreviations: Adaline: Adaptive Linear Element; KNN: K‐Nearest Neighbor; BP: Back Propagation; CART: Classification and Regression Tree; SVM: Support Vector Machine; LASSO: Least Absolute Shrinkage and Selection Operator; KPCA: Kernel Principal Component Analysis; GBM: Gradient Boosting Machine; LLE: Locally Linear Embedding; ISOMAP: Isometric Feature Mapping; t‐SNE: t‐distributed Stochastic Neighbor Embedding; XGBoost: eXtreme Gradient Boosting; MLP: Multi‐Layer Perceptron; LSTM: Long Short‐Term Memory; DBN: Deep Belief Net; VAE: Variational Autoencoder; GAN: Generative Adversarial Network; ResNet: Residual Neural Network; DenseNet: Dense Convolutional Network; FCN: Fully Convolutional Network; ViT: Vision Transformer; SENet: Squeeze‐and‐Excitation Network; Faster RCNN: Faster Region‐based Convolutional Network. AI, artificial intelligence.

Artificial intelligence (AI), formally established at the 1956 Dartmouth Conference as a branch of computer science, aims to simulate the process of human learning and memory and make machines intelligent.^2^ The improved computing power makes the training of AI algorithms highly feasible and efficient, and an increasing amount of clinical big data can be used for training. Machine learning (ML), a branch of AI, is a method to train a model through past experience (input training data) and then use it for prediction.^3^ The limitation of traditional ML is that researchers need to process raw data as structured data with artificially selected features, which limits its application in complicated environments such as medical diagnosis. Thus, feature engineering is an imperative step in ML, which directly affects the accuracy of the predictions. DL is an ML method that can automatically learn and extract multilayer features, rather than handmade shallow features, from a large amount of raw data. As DL has been sufficiently shown to be able to accurately find very deep and abstract features, this has made it a widely researched topic in the field of medical image analysis. With advances in computational power, DL‐based AI has revolutionized various fields. The historical development of AI models is illustrated in Figure 1.

In this review, DL and its applications in precision medicine and glioma are explored. We focused on DL developments in glioma, discussed their insufficiency and solutions, and proposed directions for future research.

## 2 SUBTYPES OF ML AND DL

DL has been successfully applied in various fields such as speech recognition, text translation, automatic driving, and object detection. In medical diagnosis, DL helps doctors solve many tasks. Its vast application fields are unprecedented compared to other technologies. This can mostly be attributed to the different subtypes and architectures of ML and DL that render DL various features and make DL fit for multiple tasks.

**FIGURE 2:**
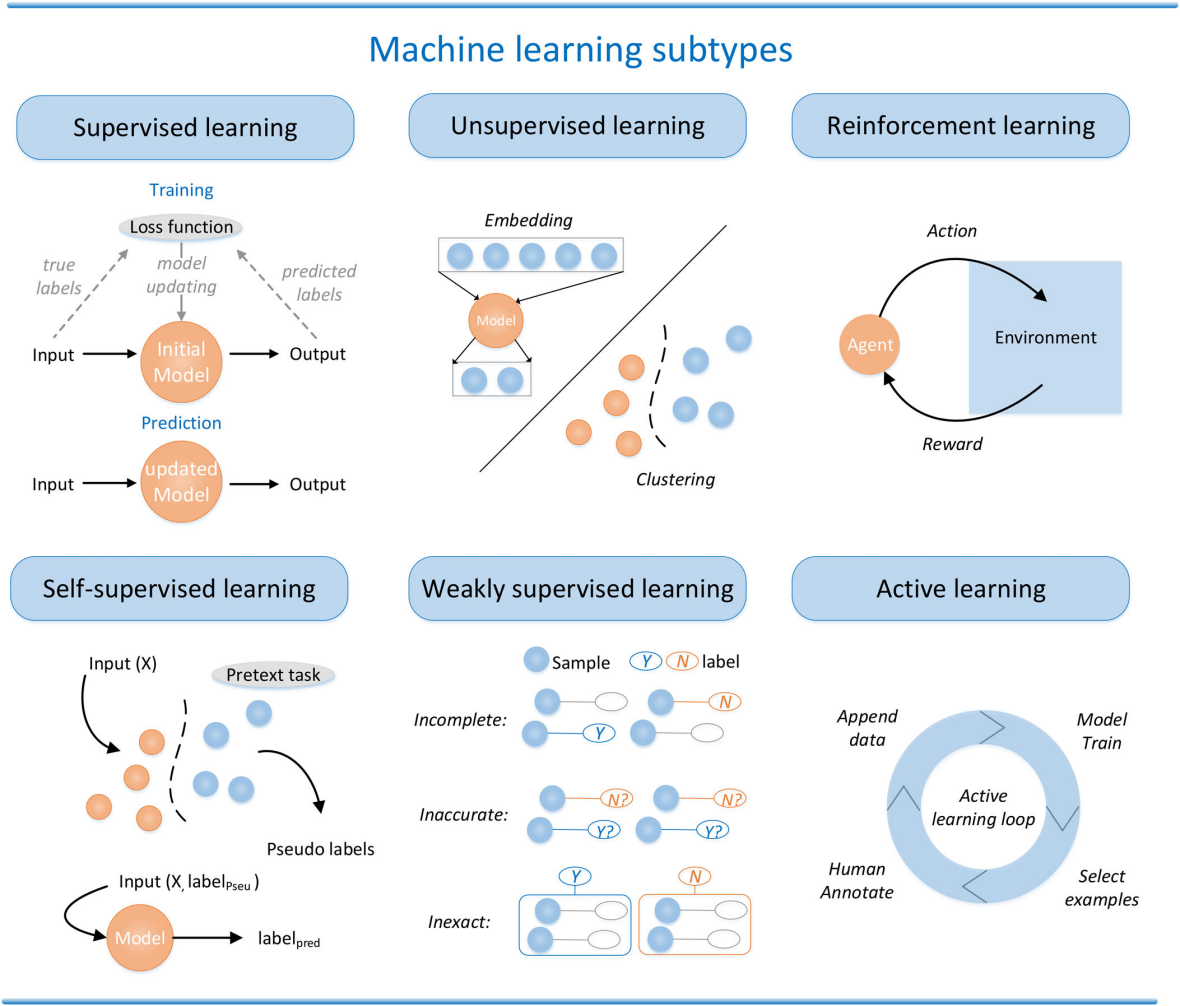
Different types of ML. Supervised learning: training process is shown above and prediction process is shown below. Unsupervised learning: the main two applications—embedding and clustering. Reinforcement learning: learn from environment and update from feedback. Self‐supervised learning: a pretext task in self‐supervised learning is a task designed to train a neural network to learn useful representations of input data without explicit supervision. The network is trained to solve the pretext task using the input data as the only source of supervision, and the learned representations can be transferred to downstream tasks where explicit supervision is available. Weakly supervised learning: the main three weak supervision types are incomplete supervision (some examples lack label), inaccurate supervision (some labels are wrong), and inexact supervision (label strength is weak, such as classification labels compared to segmentation labels). Active learning: the main four processes—selecting examples, annotation, appending data, and model training—are iteratively implemented and form a loop.

There are several subcategories of ML,^4^, ^5^, ^6^, ^7^ including supervised, unsupervised, reinforcement, self‐supervised, weakly supervised, and active learning. Figure 2 presents a detailed illustration of these six subcategories.

*Supervised learning*: This method is a machine‐learning task for inferring functions from a labeled training dataset.^8^ As the most common ML method, the supervised learning algorithm analyzes the training data, and then the trained network and optimized parameters can be used to map new samples based on the training data and generate prediction outputs that can determine the class labels of the new inputs. The main tasks that use supervised learning include solving classification and regression problems. The training, validation, and test process in supervised learning involves splitting the labeled dataset into a training set, a validation set, and a test set. The training set is used to train the algorithm by iteratively adjusting the model's parameters to minimize the error between the predicted output and the actual output. The validation set is used to evaluate the model's performance during the training process. And the test set is used to evaluate the model's performance on new, unseen data. This helps prevent overfitting and provides an unbiased estimate of the model's performance. Li Y et al.^9^ assessed several supervised ML methods such as support vector machine (SVM), random survival forest (RSF), tree gradient boosting (GB), and component GB's performances for glioma patient survival prediction after tumor resection. The models were trained by clinical characteristics and biomarker information of 776 glioma cases; results showed that the GB model with concordance index of 0.84 performed better than others.

*Unsupervised learning*: This method can be applied when there is insufficient prior knowledge, such as when it is difficult to label categories manually or when the cost of manual category labeling is too high. The unsupervised learning network does not provide standard data samples with correct class labels initially. Unlike supervised learning, the original unlabeled dataset is directly integrated into the network so that the network learns by itself according to the data characteristics. The main tasks that use unsupervised learning include clustering and dimensionality reduction. Leelatian et al.^10^ proposed an unsupervised and automated unsupervised and automated ML algorithm for Risk Assessment Population IDentification called RAPID, which uses mass cytometry dataset of 2 million cells from 28 glioblastomas as input and clustering the data into 43 cell clusters, and RAPID identifies specific cell clusters that link to clinical outcomes and stratify patient survival.

*Reinforcement learning*: Reinforcement learning (RL)^11^ theory differs from supervised and unsupervised learning in that RL does not require any data to be given in advance but obtains learning information and updates model parameters by receiving feedback from the environment on actions. RL involves an agent that interacts with an environment by taking actions and receiving rewards, with the goal of learning a policy that maps states to actions that maximizes the expected cumulative reward over time. The agent learns by trial‐and‐error through exploration of the environment, and its policy is updated based on the rewards received from the environment. RL has found applications in a wide range of domains, including robotics, recommendation systems, and control systems. Yazdjerdi et al.^12^ proposed a novel model‐free method based on RL—Q‐learning algorithm, which is developed to design a closed‐loop controller for anti‐angiogenic drug dosing by using different values of the maximum drug dosage as input and receiving feedback of tumor.

*Self‐supervised learning*: Self‐supervised learning (SSL) is a ML approach where an algorithm uses the inherent structure of the data to generate pseudo‐labels, allowing the algorithm to train itself. The algorithm learns from the data by breaking it down into smaller parts and predicting certain aspects of those parts. There is a subtle difference between unsupervised learning and SSL. In unsupervised learning, an algorithm learns to find patterns and relationships in data without any labels or guidance, whereas in SSL, the algorithm creates its own labels using the structure of the data. Multi‐omics data gained by Next Generation Sequencing significantly enrich patients' information, but inter‐omics relationships on unlabeled multi‐omics data still lack methods to exploit. Hashim et al.^13^ proposed a novel pretraining paradigm that consists of various SSL components which are capable of learning inter‐omics relationships to achieve contrastive alignment, data recovery from corrupted samples, and recovery of one type of omics data using other omic types. The pretraining paradigm based on SSL greatly improves performance on downstream tasks with limited labeled data.

*Weakly supervised learning*: Weak supervised learning is a ML technique that involves training models using partially labeled data, as opposed to fully labeled data. In weak supervision, instead of providing precise labels for every data point, only partial, incomplete, or noisy labels are used to train the model. This approach is useful when obtaining fully labeled data is costly, time‐consuming, or simply not possible. There are three common weak label types including incomplete labels, inaccurate labels, and inexact labels as Figure 2 shows. Decision support systems for pathology had been hindered by the large need of manually annotated datasets. Campanella et al.^14^ presented a multiple instance learning‐based DL workflow, which belongs to the inexact labels situation in weakly supervised learning. The system was evaluated on a dataset of 44,732 whole slide images (WSIs) from 15,187 patients and results showed AUC values over 0.98 for all tested cancer types and thus proved its great potential in clinical practice.

*Active learning*: Active learning is a ML technique that involves iteratively selecting the most informative data points to label based on uncertainty or model confidence, and requests human annotation for these examples. The goal of active learning is to reduce the amount of labeled data needed to train a model while maintaining or improving its performance. Therefore, they are used in tasks with high labeling costs, such as medical imaging. Hao et al.^15^ proposed a novel transfer learning‐based active learning framework, which can reduce the annotation cost and maintain the model performance for brain tumor classification in the meantime.

DL technology as a subcategory of AI can solve various tasks by building deep neural networks (DNNs).^16^ An important distinguishing characteristic of DL compared with other AI subcategories is its self‐learning ability. All features of the representation learning process in DL are automatically learned from the input training data. This characteristic makes DL perform better at self‐optimizing models for specific problems than artificial feature engineering approaches.^17^, ^18^

**FIGURE 3:**
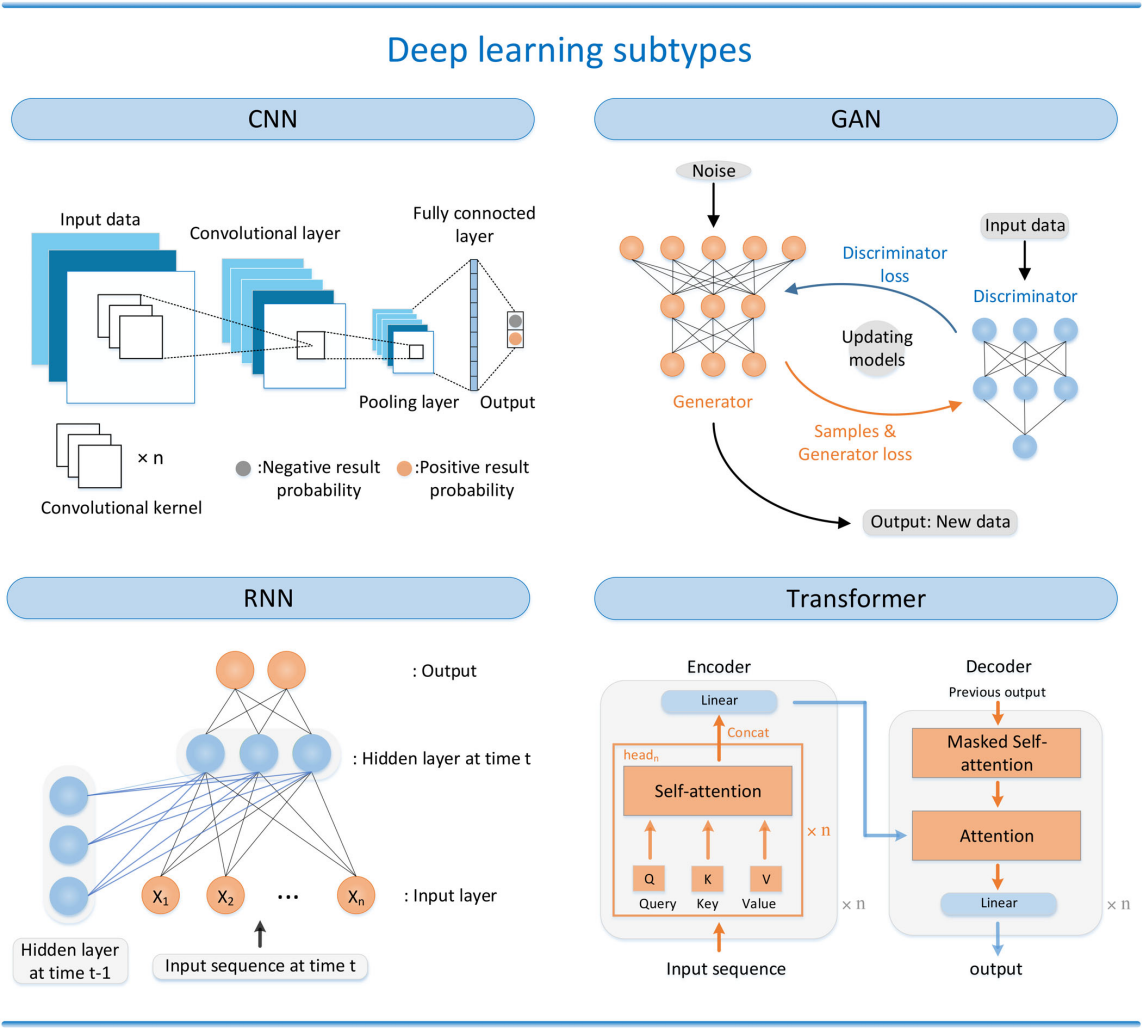
Different structures of DL. CNN: the main three components from input data to output in a CNN—convolutional layer, pooling layer, and fully connected layer. GAN: the main two components in a GAN—a generator using noise as input and a discriminator using real data as input. RNN: connecting hidden layer with previous values. Transformer: a transformer can be divided into two parts—encoders and decoders, compared with other structures, a transformer is completely based on the attention mechanisms. CNN, convolutional neural network; DL, deep learning; GAN, generative adversarial network; RNN, recurrent neural network.

A layer is a fundamental building block of a DNN. In a DNN, layers are stacked one after the other to form a network architecture. Each layer consists of a set of neurons, which are connected to the neurons in the previous and next layers by different weights that can be trained. The input layer is the first layer of the network, which receives the input data. The output layer is the last layer of the network, which produces the final output. In between the input and output layers, there can be one or more hidden layers, which are responsible for extracting useful features from the input data and transforming them into a form that can be used to make predictions. A DNN contains multiple hidden layers. In addition, various types of DNN models exist, such as convolutional neural networks (CNNs), recurrent neural networks (RNNs), generative adversarial networks (GANs), and transformers. Figure 3 shows these main DL structures.

Among these, the CNN^19^ model is the most widely used, whose process of recognizing, processing, and analyzing image features is similar to the process of processing visual information by the human nervous system.^20^ A CNN is characterized by local connection, weight sharing, pooling operation, and multilayer structure. The convolutional layers in the CNN automatically extract features of the input information and reduce the complexity of the model through weight sharing. This is followed by the pooling layer, which obtains spatially invariant features by reducing the resolution of the feature maps.

RNN,^21^ through its recurrent structure, is aimed at learning temporal correlations and is suitable for performing inference on temporal sequence data. The recurrent layer is the key component that distinguishes RNNs from other types of neural networks. It contains a set of recurrent connections that allow the network to store information about the previous time‐steps and use it to make predictions for the current time‐step. This makes RNNs particularly effective for modeling time‐series data or any other type of sequential data where the current input is dependent on the previous inputs.

The GAN^22^ architecture can simultaneously train a generator and discriminator, which enables the GAN to obtain new samples from the input training data distribution. The generator network takes random noise as input and is trained to create samples that are as close as possible to real data, to fool the discriminator into classifying them as real. The discriminator network, on the other hand, takes true data as input and outputs a probability that the sample is real. The discriminator is trained to distinguish between real data and fake data samples generated by the generator network and provide feedback to the generator on how to improve the quality of the generated samples. The generator and discriminator are trained in an adversarial manner, and this process is repeated many times until the generator is capable of generating realistic data samples. GANs have been used in various applications, such as image and text generation, style transfer, and data augmentation.

The Transformer^23^ is a DL model that was introduced in 2017 for natural language processing (NLP) tasks. At a high level, the basic structure of a Transformer consists of an encoder and a decoder. The encoder is made up of multiple identical layers, each of which has two sub‐layers: a self‐attention layer and a feedforward layer. The self‐attention layer allows each token in the sequence to attend to all other tokens, which helps capture long‐range dependencies in the input. The feedforward layer then applies a nonlinear transformation to each token's representation. The decoder is also composed of multiple identical layers, each of which has three sub‐layers: a masked self‐attention layer, an encoder‐decoder attention layer, and a feedforward layer. The masked self‐attention layer allows each token in the output sequence to attend to all previous tokens. The encoder‐decoder attention layer allows the decoder to attend to the encoder's output and thus incorporate information from the input sequence. To summarize different types of ML and DL structures, ML and DL subtypes' key features and major uses are included in Table 1.

**TABLE 1:** Comparison table of different ML subtypes and DL structures.

ML subtypes	Key features	Major use
Supervised learning	Learns from input‐output pairs to predict or classify new input data	Classification and regression
Unsupervised learning	Input data without corresponding labels, learns to discover patterns in the data on its own	Clustering, dimensionality reduction, and anomaly detection
Reinforcement learning	Make optimal decisions by interacting with an environment	Robotics control and autonomous driving
Self‐supervised learning	Uses the characteristics of the data itself for supervision	Image, video, and speech recognition
Weakly supervised learning	Incomplete/inaccurate/inexact data labels	Image and speech recognition
Active learning	Representative samples are selected for annotation to better train the model	Image and speech recognition

DL structures	Key features	Major use
CNN	Convolutional layers and pooling layers, which can effectively extract features from images	Image and speech recognition
RNN	Recurrent layer, which can process data with front and back correlation	Natural language processing, speech recognition
GAN	Two neural networks, a generator and a discriminator, which generate realistic samples through adversarial training	Image generation, video generation
Transformer	Self‐attention mechanism and multi‐head attention mechanism, which can process long text sequences	Machine translation, text generation, question answering

Abbreviations: CNN, convolutional neural network; DL, deep learning; GAN, generative adversarial network; ML, machine learning; RNN, recurrent neural network.

## 3 DL IN PRECISION MEDICINE

**FIGURE 4:**
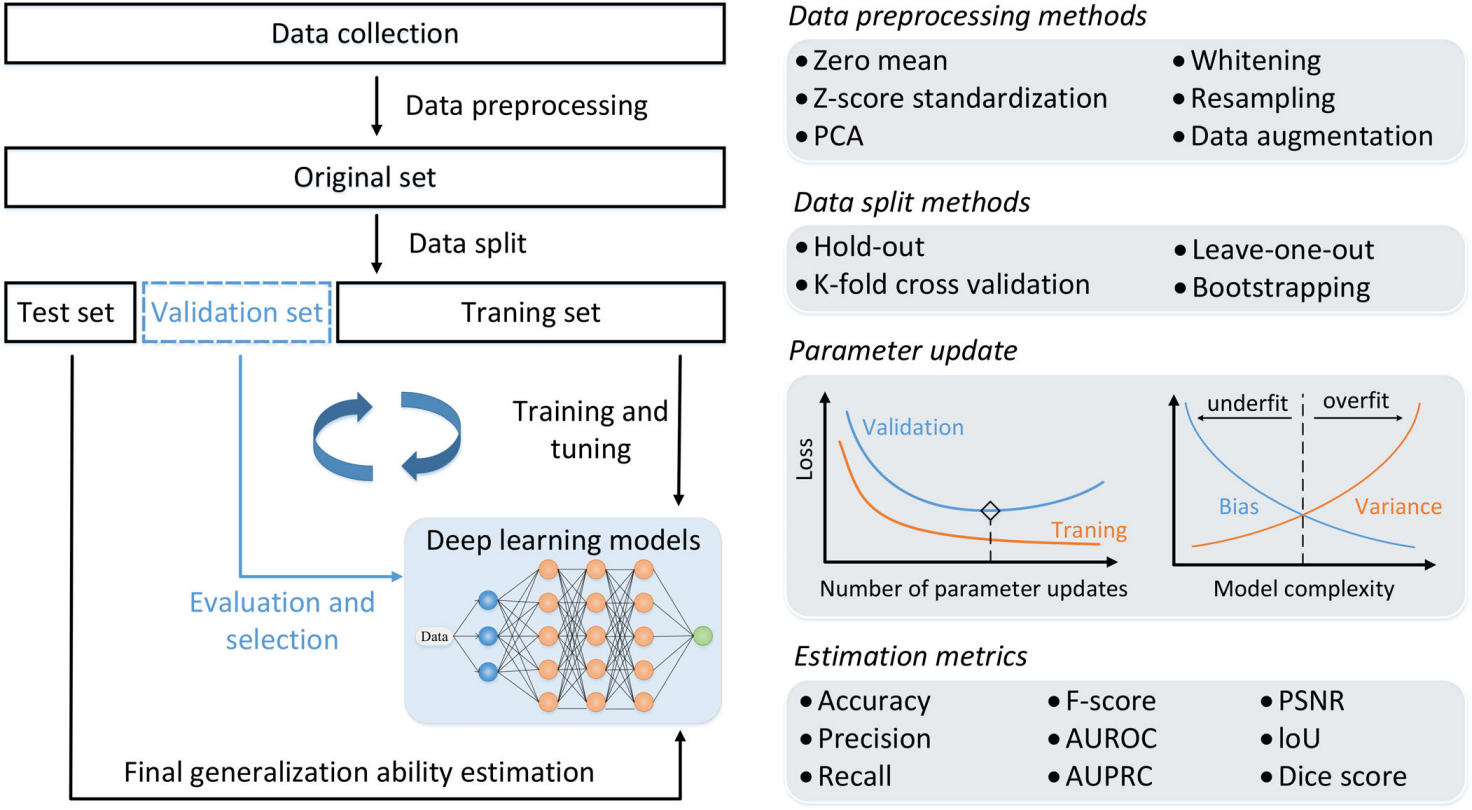
Workflow of DL in precision medicine. Left part shows an overall and basic process of deep learning model training, validation, and test. And right part shows corresponding methods and features in the left part such as data preprocessing, data split, parameter update, and estimation. DL, deep learning.

In precision medicine, DL algorithms can be used to analyze large and complex datasets, such as genomic data, electronic health records, and medical imaging data, to identify patterns and make predictions about disease risk, treatment response, and patient outcomes. The basic steps and strategies for DL workflow in precision medicine are relatively similar, as shown in Figure 4.

Data collection is the first critical component in the DL workflow and determines the upper limit of its performance to some extent. To ensure that DL models are trained on the high‐quality dataset and that the dataset is suitable for training, various data preprocessing, such as zero mean, whitening, Principal Component Analysis (PCA), and data augmentation, can be used. Zero mean: This method is useful in cases where the data has a significant bias, as it helps to center the data around zero. Whitening: This is a technique used to decorrelate the data by transforming it into a new space where the covariance matrix is the identity matrix. PCA: This is a technique used to reduce the dimensionality of the data by identifying the principal components, which are the directions in the data with the highest variance. Data augmentation: This technique involves generating new training data by applying various transformations to the original data, such as flipping, rotating, or scaling the images. This can help to increase the size of the training set and improve the robustness of the model to variations in the input data.

Then, the original dataset must be split into several parts as requirements, which often will be split into a training set and test set. And there are several commonly used data split methods such as hold‐out, leave‐one‐out, bootstrapping, and K‐fold cross validation. Hold‐out: This method splits data into two disjoint subsets, usually in a 7:3 or 8:2 ratio, with one subset used for training the model and the other for testing its performance. Leave‐one‐out: This method involves using a single data subset for testing and the remaining data subsets for training. This is repeated for all data subsets, and the average performance across all iterations is used as the final evaluation metric. Bootstrapping: This method randomly selects data subsets from the dataset with replacement to create multiple training and testing sets. This is useful when the dataset is small and the goal is to get a more accurate estimate of the model's performance. K‐fold cross‐validation: This method involves dividing the dataset into *K* equal‐sized subsets, where *K* − 1 subsets are used for training and the remaining subset is used for testing. This is repeated *K* times, with each subset used exactly once for testing. The average performance across all iterations is used as the final evaluation metric. *K* is usually set to 5 or 10.

Furthermore, sometimes it needs the validation set to evaluate the training results and select the best model to avoid underfitting or overfitting of the training set while updating its parameters in models. Overfitting and underfitting are common problems that can occur when training ML models. Overfitting occurs when a model is too complex and learns the training data too well, to the point where it begins to memorize the training data instead of learning the underlying patterns. As a result, the model may perform very well on the training data, but poorly on new, unseen data. Overfitting can lead to poor generalization, where the model is not able to perform well on new data. Underfitting, on the other hand, occurs when a model is too simple and is not able to capture the underlying patterns in the data. This can lead to poor performance on both the training and test data.

To minimize overfitting, several techniques can be used, such as: Regularization: Adding a regularization term to the loss function of the model can help to penalize complex models, and prevent them from overfitting. Dropout: Dropout is a technique that randomly drops out some neurons during training, which can help to prevent the model from overfitting by reducing its capacity. Early stopping: Monitoring the validation loss during training and stopping the training when the validation loss starts to increase can help to prevent overfitting. Data augmentation: Increasing the amount of training data by applying transformations to the existing data, such as rotating or flipping images, can help to prevent overfitting.

To minimize underfitting, several techniques can be used, such as: Increasing model complexity: Adding more layers or neurons to the model can increase its capacity and help it to capture more complex patterns in the data. Feature engineering: Careful feature selection or extraction can help the model to capture more relevant information from the data. Adding more training data: Increasing the amount of training data can help the model to learn more about the underlying patterns in the data.

And the model was better trained on multiple centers datasets and tested on an external independent dataset to ensure that the model generalizes well. A single dataset may have inherent biases that can be reflected in the model; multiple centers research ensures that the results of a study are not dependent on the specific conditions of a single research center. This increases the reproducibility of the results and strengthens the confidence in the model. The goal of DL is to develop models that can generalize well to new data. To ensure that a model is truly generalizing, it needs to be validated on an external dataset that it has not been trained on. For example, in a study using patients' metabolomic state to predict diseases outcomes over 110,000 people,^24^ researchers trained their DL models on 22 recruitment centers and performed external validation in four independent cohorts to assess their models' clinical performances. There are several commonly used medical datasets which can benefit DL modeling. Their dataset range and major use are listed in Table 2.

**TABLE 2:** Introduction of multiple datasets.

Dataset name	Dataset range	Major use
**Medical images datasets**		
TCIA	Over 1.8 million multi‐modal images from 35,000+ subjects, 170+ collections	Cancer detection, diagnosis, and treatment
MURA	14,000+ musculoskeletal x‐rays	Classification of normal and abnormal bone images
ISIC	23,000+ images of skin lesions	Skin lesions detection
ChestX‐ray8	100,000+ chest x‐rays	Classification of eight common thoracic diseases
BraTS	MRI images from 393 cases of glioma	Brain tumor segmentation and recognition
COVID19‐CT	1000+ chest CT images of patients with confirmed COVID‐19 diagnosis	COVID19 detection and diagnosis
**Electronic health record (EHR) datasets**		
MIMIC‐III	40,000+ patients with demographic, clinical, and outcome data	Patients' outcome prediction and diseases risks assessment
eICU	200,000+ ICU patient records	Patients' survival prediction
UK Biobank	500,000+ individuals with demographic, lifestyle, and health data	Develop methods for disease prevention, diagnosis, and treatment
**Omics dataset**		
TCGA	11,000+ patients with cancer across 33 different cancer types	Identify potential targets for new therapies, and develop predictive models for patient outcomes
PDB	170,000+ protein structures from organisms	Prediction of protein structure, design new drugs and therapeutic agents
KEGG	22,000+ human genes, 600+ diseases, and associated molecular pathways	Explore functional relationships between genes, proteins, and other molecules
HMDB	114,000+ metabolites' structures, functions, and associated diseases	Identify potential biomarkers for diagnosis and treatment of various conditions

Abbreviations: BraTS, Brain Tumor Segmentation Challenge; eICU, eICU Collaborative Research Database; HMDB, Human Metabolome Database; ISIC, International Skin Imaging Collaboration; KEGG, Kyoto Encyclopedia of Genes and Genomes; MIMIC‐III, Medical Information Mart for Intensive Care III; MURA, Stanford's Musculoskeletal Radiographs; PDB, Protein Data Bank; TCGA, The Cancer Genome Atlas; TCIA, The Cancer Imaging Archive; UK Biobank, UK Biobank Imaging Study.

The final critical step in DL workflow is the assessment of its performance. The area under the receiver operating characteristic curve (AUROC/AUC) achieves a trade‐off between sensitivity and specificity, which is often used to assess the performance of DL models in classification tasks. Typically, AUC > 0.80 is considered good for most tasks; however, whether this threshold is suitable for clinical use still needs comprehensive consideration. This is also an important reason DL is not yet fully accepted by clinicians in hospitals. A good classifier should achieve both high sensitivity and specificity. However, for some applications, it may also be important to emphasize either of them. When the model focuses on detecting positive class samples in the dataset, it is recommended to use the area under the precision‐recall curve (AUPRC), instead of the AUROC. There are also several metrics for different tasks, such as PSNR (peak signal‐to‐noise ratio) for super‐resolution and IoU (intersection over union) for object detection.

**FIGURE 5:**
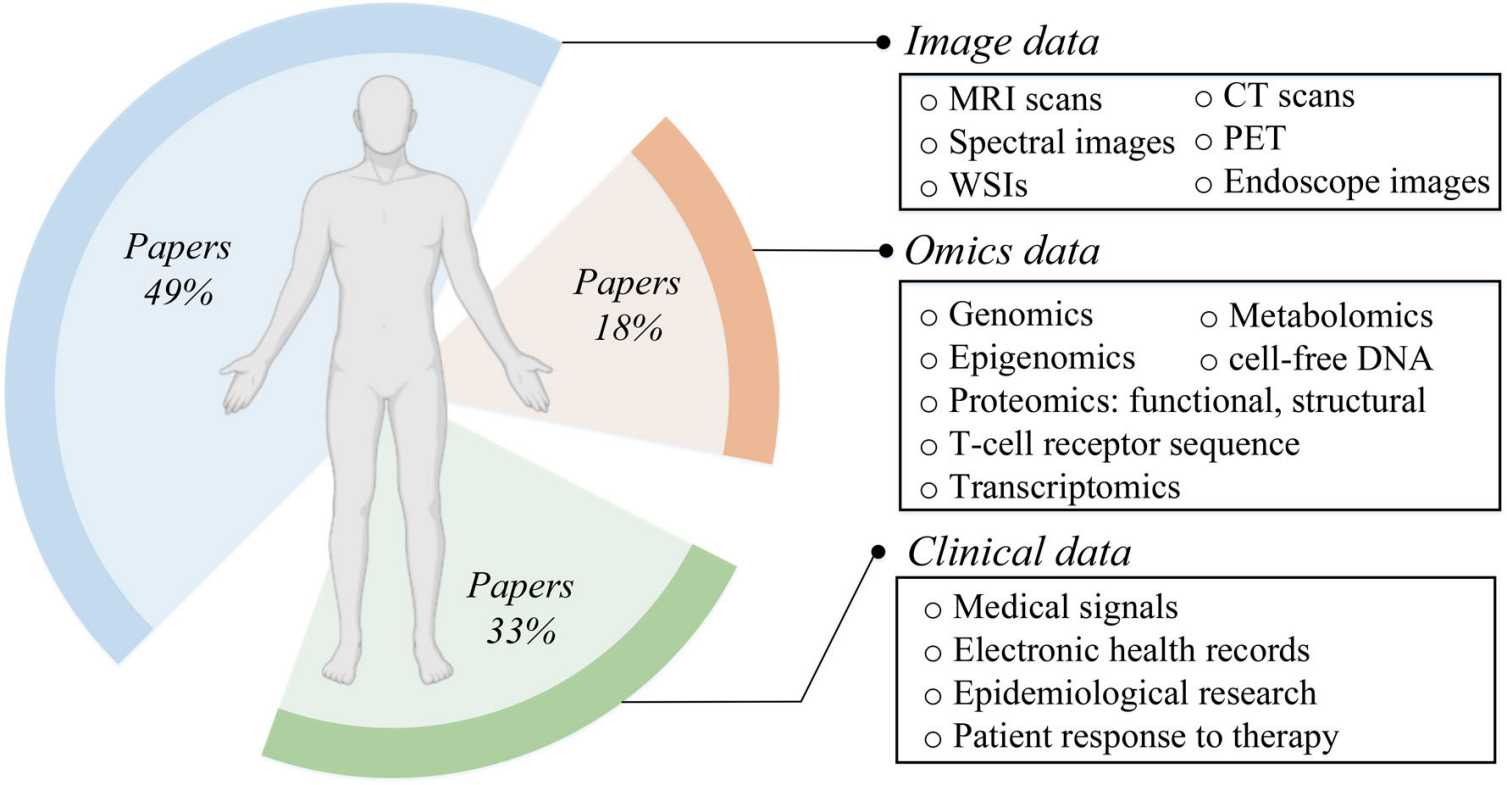
Body features and their percentages in DL. Left part shows percentages of the researches publications amounts using image data, omics data, and clinical data. Right part shows different detailed features in the datasets. DL, deep learning.

Many large cancer datasets have been established in recent years; these datasets are often used to build DL models that can assist in research and clinical decision‐making. These datasets include a large number of data types spanning genomics, epigenomics, proteomics, histopathology, and radiology images. These complex information networks have driven the development of DL in diseases. Varieties of patient data can be useful features for DNN models. Figure 5 illustrates the dataset and its features.

Image data, which includes 13,023 papers using strings “deep learning” and “medical image” in Pubmed, are the largest part of body features that can be used in DL. The second part comprise clinical data features including a total of 8652 papers using strings “deep learning” and “medical signals” (1709), “deep learning” and “record” (3951), and “deep learning” and “response” (2992) in Pubmed. The last part comprises omics data including a total of 4940 papers using strings “deep learning” and “omics” (488), “deep learning” and “genomics” (2948), “deep learning” and “proteomics” (461), “deep learning” and “T‐cell receptor sequence” (37), “deep learning” and “transcriptomics” (676), “deep learning” and “epigenomics” (128), “deep learning” and “metabolomics” (165), and “deep learning” and “cell‐free DNA” (37) in Pubmed. These researches were conducted on various diseases and medical conditions and fewer papers amounts may mean that more experiments and data collection are required, thus taking longer to complete and lead to a lower number of papers published.

### 3.1 Cardiology

The main vehicle for combining DL with cardiology is electrocardiogram (ECG), a method commonly used by cardiologists for disease risk assessment of a patient's cardiovascular system. The original approach used rule‐based ML algorithms for ECG processing, but this approach exhibited high inaccuracy.^25^ Since the rise of DL, researchers have used the superior performance of DL models to launch a series of applications in the field of cardiology research, such as using ECG to classify arrhythmia, classify 12 heart rhythm categories, diagnose atrial septal defects, and detect long QT syndrome. Here are some notable examples.

In problems of classifying arrhythmia, Murugesan et al.^26^ contrasted three deep‐learning methods, which showed that the method combining CNN with LSTM^27^ called ECGNet performed the best. (LSTM network is one of the most commonly used RNNs. Establishing a self‐loop enables networks to maintain the gradient flow for a long period.) Furthermore, they demonstrated that ECGNet can be adapted to different cardiology problems by retraining only last three layers, suggesting that transfer learning has great potential for ECG processing. In classifying 12 heart rhythm classes, the DNN model^28^ also achieved an AUC of 0.97 and its average F1 score, which is the harmonic average of precision and recall with a maximum of 1 and a minimum of 0, was 0.837 surpassing that of cardiologists with an average score of 0.780. The heart murmur caused by atrial septal defects is faint and hard to detect. Mori et al.^29^ reported the use of DL model, comprising a CNN and LSTM, to diagnose atrial septal defects using 1192 ECGs of 728 participants as model input, achieving an accuracy of 89%, a figure significantly higher than that of cardiologists (58%). QT interval prolongation can predispose to ventricular arrhythmias and sudden cardiac death. A study by Giudicessi et al.^30^ showed that using 1.6 million 12‐lead ECGs from 538,200 patients as input the DNN can detect long QT syndrome with an AUC value of 0.97, which is far better than the common ECG machines on the market and thus provide a cost‐effective method for diseases screening. These researches above showed that DL has become a reliable tool that can assist diagnosis in cardiology.

ECG can also indicate the occurrence and development of other diseases; cardiac autonomic dysfunction begins in the early stages of idiopathic Parkinson's disease (IPD). By developing a CNN consisting of 16 layers with Bayesian optimization and using 751 IPD patients (2138 ECGs) and 751 non‐IPD patients (2673 ECGs) as input,^31^ the AUROC of the model for detection of IPD was 0.924 (95% CI, 0.913–0.936) in the test set. As such early screening of IPD can be clinically feasible with the help of DL and establishing reliable DL/AI models can be an important step forward toward precision and personalized medicine.

### 3.2 Gastroenterology

With the help of AI, the intelligent technology is expected to solve the problems that gastroenterologist is currently facing, such as the large demand for endoscopy and uneven quality of inspections. The 5‐year survival rate of patients with advanced esophageal cancer and advanced gastric cancer is less than 20%, and the 5‐year survival rate of patients with early‐stage esophageal cancer and gastric cancer is more than 80%.^32^, ^33^ Early diagnosis and treatment are key to improving the prognosis of patients with gastrointestinal cancer.

The response of patients with gastrointestinal cancer to immunotherapy is greatly influenced by microsatellite instability (MSI), but not all patients are screened for MSI in clinical practice as it involves additional genetic or immunohistochemical tests. To fill this gap, Kather et al.^34^ demonstrated that DL can directly predict MSI from H&E histology, which is widely accessible. Study results showed that DL with H&E slides as input has great promise of expanding MSI screening to a larger group of patients with gastrointestinal cancer.

Gastrointestinal endoscopy is a popular field of medical AI research. Zhang M et al.^35^ used white‐light endoscopy (WLE) images as a training set and obtained three CNN‐based models for the tasks of differential diagnosis of benign esophageal protruded lesions, which can be used to distinguish esophageal leiomyoma, esophageal cyst, and esophageal papilloma, thus helping to improve the precision level of endoscopists' diagnosis. Ling et al.^36^ built a real‐time CNN system that can accurately identify the differentiation status of early gastric cancer and outline the margins of early gastric cancer in magnifying narrow‐band images with an accuracy of 83.3% and 82.7%, respectively, which can provide an aid for endoscopic treatment of early cancer. In the early gastric cancer detection study of Tang D et al.,^37^ 21,785 narrow band imaging endoscopy images and 20 videos from five centers were chosen and trained a YOLOv3^38^ AI system, a high‐precision single stage object detection algorithm that can meet real‐time detection requirements (FPS > 30), and achieved a diagnostic accuracy of 93.2%, which is better than senior endoscopists (85.9%). Multi‐center researches proved that DL models have significant application capabilities in gastrointestinal diseases, which can greatly promote the development of precision medicine in this field, and provide a favorable tool for early screening and discovery of patients with gastrointestinal disease risks.

### 3.3 Ophthalmology

The first paper on the application of AI to the screening of diabetic retinopathy was published in 2016,^39^, ^40^ which opened up the path for the application of AI in ophthalmology. Since then, AI has shown great application prospects in various fields of ophthalmology, such as fundus disease, glaucoma, cataract, myopia, corneal disease, and orbital disease.^41^ AI analysis of imaging results, such as fundus tomography, optical coherence tomography imaging (OCT), and fluorescein fundus angiography, can assist in the screening, diagnosis, grading, and guiding of treatment.^42^

Keenan et al. trained a DNN called DeepLensNet^43^ on the Age‐Related Eye Disease Study (AREDS) dataset, which includes information on 4757 participants with their visual function testing results, to quantitatively classify cataract type and severity and its performance was compared with that of 14 ophthalmologists and 24 medical students. The results showed that the accuracy of DeepLensNet was significantly superior to that of experts and such approaches based on DL technology can greatly enhance the accessibility of cataract assessment. And based on data such as corneal topography, anterior segment OCT, and 3D images of the Pentacam anterior segment, several research teams^44^, ^45^, ^46^, ^47^ have successively developed multiple automatic grading methods for keratoconus using CNNs, with an accuracy of 99.3%.

AI analysis based on fundus and retinal photographs can also be used to predict^48^ cardiovascular and cerebrovascular diseases. Rim et al. proposed a novel cardiovascular risk stratification system based on DL, their model called RetiCAC used 216,152 retinal photographs from five datasets as input and outperformed all single clinical parameter models in predicting coronary artery calcium, which is a marker of cardiovascular disease risk, and is also comparable to CT scan‐measured CAC. This research also implied the great potential of ophthalmology photographs being applied to wider realms and thus accelerating the process of precision medicine.

### 3.4 Dermatology

Skin tumors, a common skin proliferative disease, are clinically divided into benign and malignant tumors. Malignant skin tumors are prone to invasion of the surrounding tissues and organs and metastasis. Early diagnosis and timely treatment can improve the cure and survival rates.

Esteva et al.^49^ used 129,450 clinical skin images to train a deep CNN to diagnose skin cancer. The neural network trained in this manner can classify skin cancer comparable to the ability of dermatologists. In a study of remote diagnosis of skin cancers, Huang et al.^50^ built a light‐weight skin cancer classification model based on DL methods and proved its ability of aiding first‐line medical care. And by comparing multiple models, their results showed that DenseNet performed better on benign and malignant binary classification tasks, and EfficientNet performed better comprehensively on multiple classification tasks. The accuracy reached 89.5% and 85.8% for the binary classifications and seven‐class classification. Mijwil^51^ also tested three different DL architectures on the The International Skin Imaging Collaboration (ISIC) 2019 dataset and ISIC2020 datasets, which is a large collection for skin cancer detection containing over 33,000 images of skin lesions, including malignant melanoma, benign nevi, and seborrheic keratoses, and the final experimental results showed that Inception v3 performed better.

Khouloud et al.^52^ proposed a DL model for melanoma detection that consisted of the segmentation network W‐Net and the classification network Inception ResNet. Experiments indicate that the model has excellent segmentation and classification abilities with higher accuracy. Khan et al.^53^ also proposed a DL network, including segmentation and classification, which was tested on multiple datasets and obtained good scores. However, these models have high requirements for texture, color, and background. Nersisson et al.^54^ fused YOLO and CNN and proposed a new classification network that uses YOLO to extract focal areas before completing the classification of skin diseases using CNN. The study achieved 94% accuracy on the ISIC2016 dataset, and was largely unaffected and robust when processing images with hair and sweat. These DL models show tremendous potential to improve skin cancer research and extend skin lesions screening beyond the clinical setting, which makes medicine more personalized and precise in the future.

**FIGURE 6:**
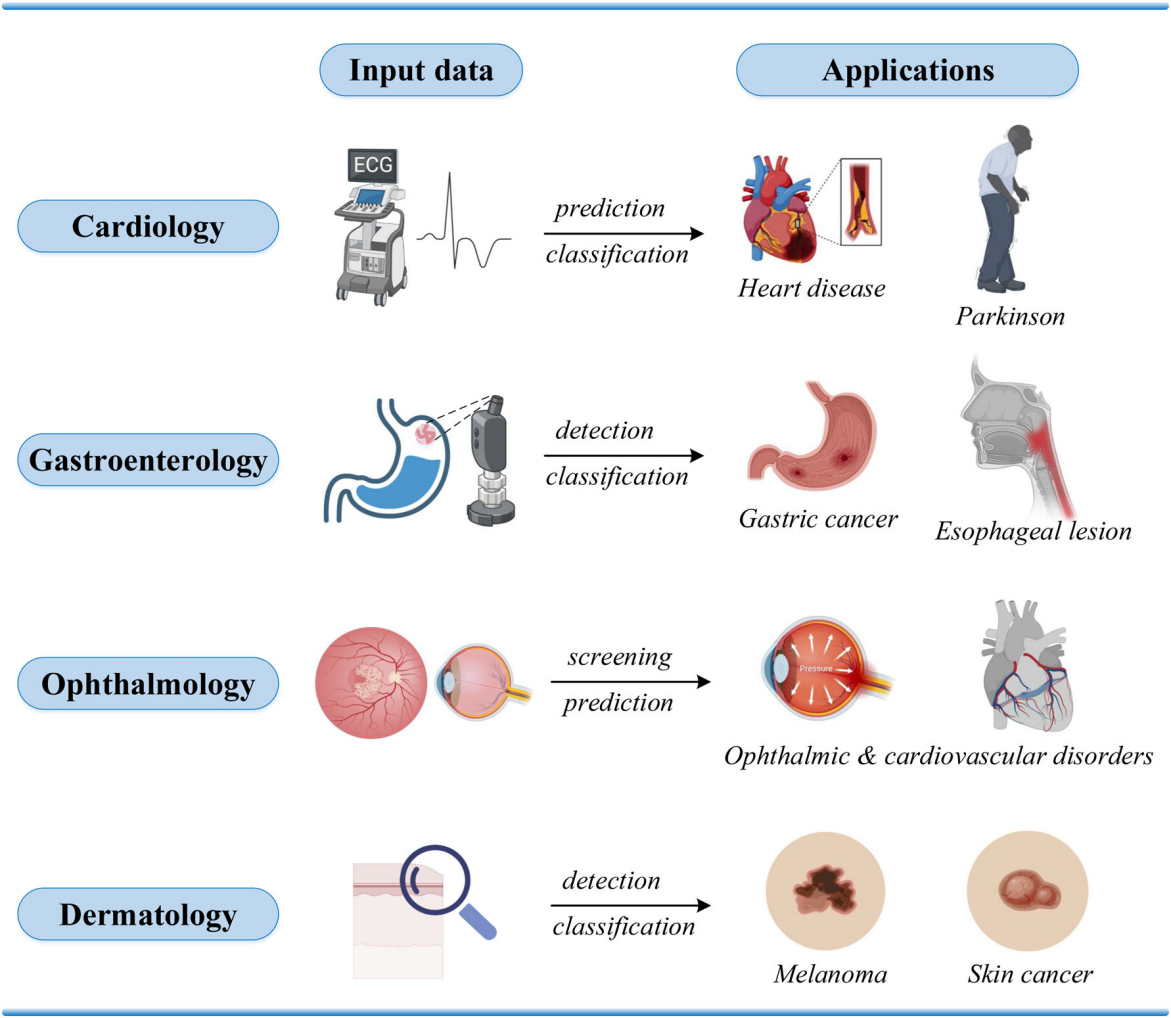
DL in clinical fields. Cardiology: applications such as predicting heart diseases and Parkinson using ECG as input. Gastroenterology: applications such as detecting and classifying gastric cancer and esophageal lesions using endoscope images as input. Ophthalmology: applications such as predicting ophthalmic and cardiovascular disorders using fundus and retinal images as input. Dermatology: applications such as classifying melanoma and skin cancer using clinical skin images. DL, deep learning.

Generally, DL shows great promise in various clinical fields according to the researches above. Its main input data and applications in different realms are summarized in Figure 6. DL models can help clinicians tailor treatments to individual patients based on their specific clinical characteristics. This can lead to more effective treatments with fewer side effects. Furthermore, DL models can analyze large amounts of medical data to detect early signs of diseases, such as cancer and Parkinson's, before symptoms appear. This can improve patient outcomes and reduce healthcare costs. There are several challenges of DL in clinical applications, such as data quality and quantity, privacy concerns, data heterogeneity, limited availability of annotated data, model interpretability, and clinical validation. DL models can also perpetuate bias and unfairness if they are trained on biased or incomplete data. In the medical field, this can have serious consequences, such as misdiagnosis and unequal access to healthcare.

## 4 DL IN ONCOLOGY AND DL IN GLIOMAS

### 4.1 DL in oncology

Cancer is the most important public health problem worldwide, and its incidence is increasing every year in China.^55^ Accurate diagnosis of various tumors is important for improving the survival rate of patients. The application of precision and personalized medicine is meaningful for the treatment of different cancer patients. DL can be used for lesion detection and tumor subtyping to obtain better and easier therapeutic plans for patients with medical images of tumors.

Ayatollahi et al.^56^ applied RetinaNet to three‐dimensional (3D) breast magnetic resonance imaging (MRI) sequences. After considering 3D morphological and dynamic information, the 3D RetinaNet they proposed achieved 90% accuracy and 95% sensitivity in detecting malignant and benign breast lesions. Liu et al.^57^ proposed a breast cancer‐detection AI system called LYNA to automatically evaluate lymph node biopsies; the algorithm they developed had achieved higher tumor‐level sensitivity than pathologists while attaining comparable slide‐level performance. Such aided diagnostic methods can improve the speed, accuracy, and consistency of a physician's diagnosis and can even reduce the false‐negative rate to 1/4 of that of a human pathologist. Abdul^58^ proposed a CNN‐based automatic lung cancer classification and detection system with an accuracy rate of 97.2%, sensitivity of 95.6%, and specificity of 96.1%. A weakly supervised CNN algorithm proposed by Xu et al.^59^ classified lung tumors with an AUC of 0.9978. Most studies have shown that DL performs well in oncology tasks.

Identifying survival subgroups of patients with cancers significantly improve patient care and clinical decision. Chaudhary et al.^60^ presented a DL‐based model on hepatocellular carcinoma (HCC) that discriminates survival subgroups of patients in six cohorts using RNA sequencing, miRNA sequencing, and methylation data from TCGA as input. This multi‐omics model was validated on five external datasets of different regions and results showed a robust mean concordance index of 0.74, which provide useful workflow for reference at cancers prognosis prediction. Poirion et al.^61^ introduced DeepProg, a novel ensemble framework of DL and ML methods that robustly predicts patient survival subgroups using multi‐omics data. DeepProg identifies two optimal survival subtypes in most cancers and yields significantly better risk‐stratification than other multi‐omics integration approaches.

### 4.2 DL in gliomas

Gliomas are the most common primary central nervous system tumors, accounting for approximately 75% of all primary malignant brain tumors in adults. For traditional classifications,^62^ the World Health Organization (WHO) classifies gliomas as low‐grade gliomas (LGG, including WHO grades I–II) and high‐grade gliomas (HGG, including WHO grades III–IV). As the most common primary brain tumor, various degrees of glioma are associated with different prognoses and suitable therapies. In mid‐2021, the WHO released the newest classification of gliomas.^63^ It first divided the diffuse glioma into adult and child types, with the adult type divided into three types: astrocytoma, isocitrate dehydrogenase (IDH)‐mutant; oligodendroglioma, IDH‐mutant and 1p/19q co‐deleted; and glioblastoma, IDH wild type.

Gliomas can be objectively classified into various subtypes based on their growth patterns, behaviors, and multiple molecular markers. There are two main methods to obtain subtype‐related information: surgery and imaging examination. Considering the cost, risk, and time factors, imaging examination is the preferred method for disease diagnosis, before, during, and after treatment. However, for precision diagnosis, pathology‐slide analysis has always been considered the gold standard for glioma classification.

**FIGURE 7:**
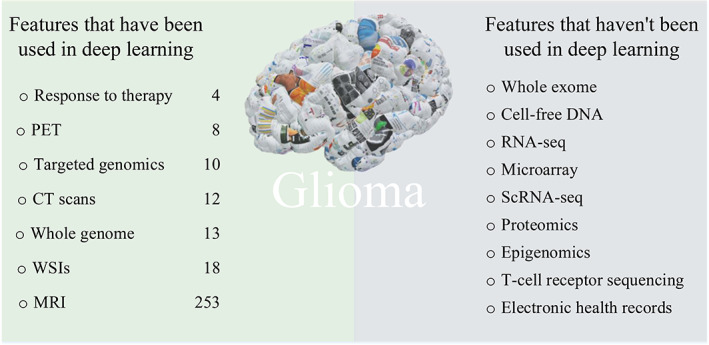
Different features in glioma used in previous studies. Collage brain image reproduced from Ref. 64. Creative Commons — Attribution 4.0 International — CC BY 4.0. Left part shows different features in glioma and their researches publications amounts. Right part shows other potential features that can be exploited in future researches.

As shown in Figure 5, multiple features can be used in different diseases. In general, any type of data that can be represented numerically can be used for training DL models. However, it is important to ensure that the data is of high quality, representative of the problems, and labeled (if possible) to enable the model to learn effectively. So the criterion for selecting training data includes the relevance of the data to the problems, the quality and consistency of the data, and the availability of the data. The formation, development, and classification of gliomas are also related to many physiological features. So these features can also have the potential to be integrated into DL algorithms. By using DNN models, features can be automatically extracted and then assist in computer‐aided diagnosis and push the development of precision medicine in gliomas. The features that have been used in previous studies and those that have not been used are listed in Figure 7.

In the left part of Figure 7, the numbers denote the number of papers using strings “glioma,” “deep learning,” and the name of the features. As shown in the figure, MRI (253 papers) and pathological images (18 papers) were the most commonly used features in past research. In glioma research, MRI and slice images are commonly used imaging tools which contain a lot of patients' clinical information and can be used to detect tumor location, shape, size, and assess the malignant degree of glioma. DL can automatically recognize and classify features in images through training on a large amount of data. Therefore, applying DL to MRI and slice image analysis can improve the accuracy of glioma diagnosis and treatment.

Multimodal medical data fusion has the potential to improve the accuracy and reliability of medical diagnosis and treatment through the use of DL techniques. However, there are several challenges that need to be addressed: *Data preprocessing*: DL algorithms require large amounts of labeled data, which can be difficult to obtain in the medical domain. Moreover, medical data can be highly heterogeneous, and preprocessing is required to ensure that data from different sources is compatible and can be integrated into a single model. *Model complexity*: DL models can be highly complex and require significant computational resources to train and optimize. Moreover, combining data from different modalities can result in highly complex models that are difficult to interpret, making it challenging to understand the underlying factors that contribute to a diagnosis or treatment recommendation. *Data quality*: The accuracy of DL models is highly dependent on the quality of the input data. In the medical domain, data quality can be highly variable due to differences in data collection methods, equipment, and protocols. *Generalizability*: DL models trained on one dataset may not generalize well to other datasets, making it challenging to apply these models in real‐world clinical settings.

### 4.3 Two main vehicles between glioma and DL

#### 4.3.1 MRI

Medical imaging is an indispensable method and assistance tool in glioma diagnosis and accounts for 90% of clinical data. Patients with suspected brain tumors need to be assessed using medical imaging, especially MRI. MRI is a noninvasive medical‐imaging technology, and it is the preferred imaging technique for the evaluation of gliomas because of its good soft tissue contrast.

MRI provides rich information for doctors to classify glioma subtypes and make appropriate treatment plans. Therefore, fully mining medical‐imaging information plays a crucial role in clinical diagnosis, decision‐making, and disease prevention. However, with the development of medical imaging technology and gradually increasing patient needs, the number of images poses great challenges to radiologists. With the rise of DL and the proposal of precision medicine, traditional subjective medical image analysis methods can no longer meet these needs, and the use of neural networks for medical image analysis has gradually become mainstream.

#### 4.3.2 Pathology

Pathological diagnosis is considered the “gold standard” for tumor diagnosis, and the results of pathological diagnosis directly affect the choice of treatment options and the prediction of prognosis. In the traditional pathological diagnosis process, the pathologist directly examines the pathological slides under a microscope and then makes a pathological diagnosis and prognosis evaluation. Hematoxylin–eosin (H‐E) staining is the most widely used histopathological slide, which clearly shows cell morphology and tissue structure. Recently, the popularization of WSIs^65^ has made the preservation and transmission of pathological slides more convenient and safe, and can better perform quantitative analysis of pathological images, thus promoting the pathology into a new development period.

Digital pathology uses the WSI digital scanning technology to obtain high‐resolution images, which successfully converts pathological tissues into high‐quality digital images. Monotonous traditional pathology diagnosis provides pathologists a heavy workload and is subjective and error‐prone when screening smears quickly. Digital pathology not only reduces the workload of pathologists, improves their diagnostic ability, and provides more valuable information but can also reduce the risk of patients with identification errors and realize the digital management of slides.^66^ Based on digital pathology, the rich dataset constructed by WSI has created the conditions for the application of DL in pathology, and integration with DL has become an important direction for the development of digital pathology.

### 4.4 Multiple tasks using DL

**FIGURE 8:**
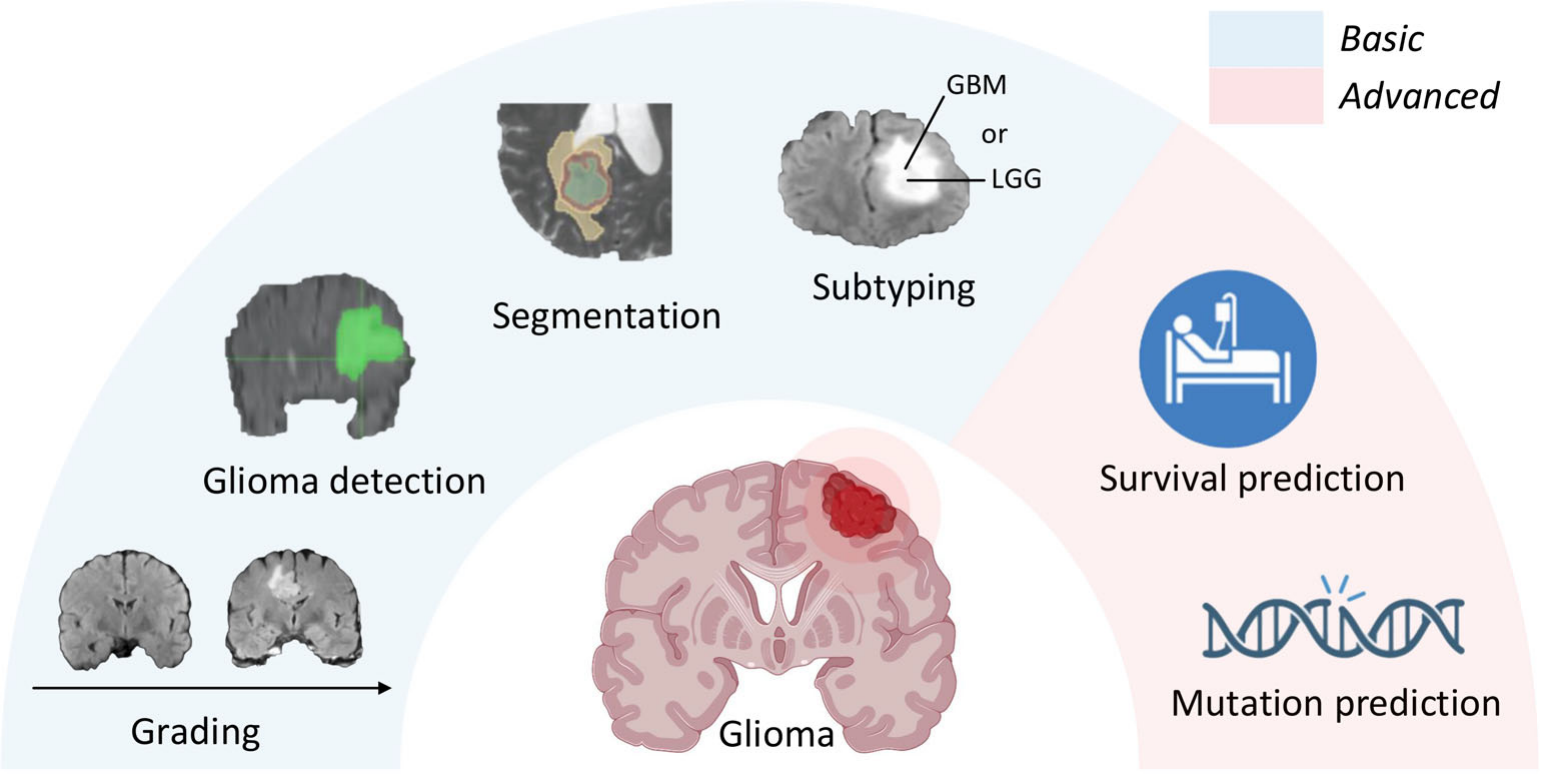
DL in gliomas. DL applications using clinical images are broadly divided into two parts—basic tasks part including gliomas grading, gliomas detection, glioma image segmentation, and glioma subtyping, and advanced tasks part including glioma patients' survival prediction and patients' genes mutation status prediction. DL, deep learning.

DL has been used for a range of tasks in gliomas, which can be broadly divided into basic and advanced tasks. Basic tasks, including tumor region segmentation, MRI image reconstruction, and glioma subtyping, mean that doctors can complete these tasks by analyzing glioma images artificially. DL only helps doctors to perform these processes in a quicker and more efficient manner, thus reducing doctors' workload and avoiding subjectivity to improve accuracy. Advanced tasks, including gene mutation prediction and survival prediction, mean that doctors cannot finish these tasks artificially only from glioma images; they require the assistance of DL. The main use of DL in the glioma field is shown in Figure 8.

It is notable that different tasks are not completely independent of each other; there are also multi‐tasks researches^67^, ^68^, ^69^ showing that different tasks' features can be shared and enhance model's performance. And the review of progresses in these tasks is as follows.

#### 4.4.1 Application in tumor segmentation

The purpose of medical image segmentation technology is to obtain clear anatomical or pathological structures in medical images. This technology significantly improves the efficiency and accuracy of diagnosis and thus plays a vital role in computer‐aided medicine.^70^ Segmentation applications for brain tumors include locating abnormal regions, automatically measuring tissue size, and computer‐guided surgery. Brain and brain tumor segmentation is a major medical image segmentation task.^71^, ^72^ Tumor segmentation generally refers to the separation of tumor tissue, such as necrosis and edema, from normal tissues, such as white matter and gray matter. Segmenting the target regions in medical images and extracting features from the segmented regions can help clinicians make more rapid and accurate diagnoses.

Image‐segmentation tasks are mainly divided into semantic segmentation and instance segmentation. However, in medical image segmentation, because each organ or tissue is very different, instance segmentation of medical images is of little significance. Thus, medical image segmentation usually refers to semantic segmentation. The goal of semantic segmentation is to accomplish pixel classification in images. Because medical picture segmentation tasks demand high accuracy, supervised learning is the most preferred approach.^73^

**FIGURE 9:**
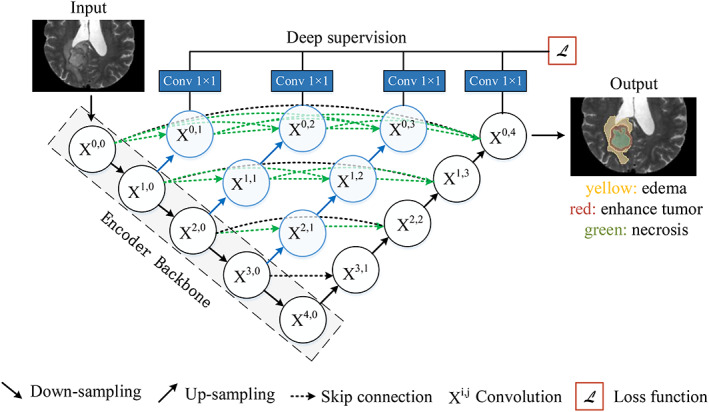
U‐Net++ structure and its application in MRI images. Up‐sampling and down‐sampling are used to adjust the size and resolution of images or feature maps. Skip connections are used to mitigate the vanishing gradient problem in deep neural networks by connecting the output of one layer directly to the input of another layer several layers away. Deep supervision is a technique that adds additional supervision signals at intermediate layers to improve the training of deep neural networks. The specific implementation of deep supervision in U‐Net++ is to add a 1 × 1 convolution kernel after X^0,1^, X^0,2^, X^0,3^, X^0,4^, which is equivalent to supervising the output of U‐Net at each level or each branch. MRI, magnetic resonance imaging.

The CNN structure called U‐Net, introduced by Ronneberger et al.,^74^ is one of the earliest algorithms for semantic segmentation using FCN, and its improved versions have been widely utilized for medical picture segmentation tasks. By merging low‐resolution and high‐resolution feature maps through skip connections, U‐Net successfully fuses low‐level and high‐level image features. There are several meaning upgrades following U‐Net. By integrating residual connections into U‐Net, Xiao et al.^75^ proposed Res‐UNet. Zhou et al.^76^ linked all U‐Net layers from one to four together and proposed U‐Net++. This architecture provides a network with the advantage of automatically learning the values of the features at different layers. The detailed structure of U‐Net++ is shown in Figure 9 and its segmentation evaluation metric, IoU has reached 89.33 using the EM dataset and 91.21 using the Cell dataset, which proved its high performance in experiences. And by cutting the number of encoder backbone layers, U‐Net++ has a born strength of network pruning which means reducing the parameters and not losing the accuracy.

From Figure 9 it can be seen that by inputting MRI images, the DL‐based segmentation algorithm has realized the automatic segmentation of gliomas into different subregions, including necrotic regions, edema regions, and tumor enhancement regions, from multimodal MRI data. Pereira et al.^77^ proposed a two‐dimensional CNN (2D CNN) automatic segmentation algorithm based on a 3 × 3 convolution kernel that uses a small convolution kernel to prevent overfitting by reducing the number of parameters. Prasanna et al.^70^ adopted CNN segmentation technology based on imaging omics, first identified the texture features of different subregions of the tumor, and then input these features into the 3D CNN (3D CNN) model for different subregions of glioma segmentation. The results showed that compared with the normal 3D CNN segmentation model, this method improved the segmentation accuracy of the tumor enhancement area and the whole tumor. Cui et al.^78^ combined two subnetworks to build a cascade of deep CNN. The segmentation results showed that the dice similarity coefficient (DSC) of the tumor, tumor core, and tumor enhancement region in the segmentation tasks were 0.89, 0.77, and 0.80, respectively. To further improve the accuracy of glioma segmentation, Mlynarski et al.^79^ used a 2D–3D CNN segmentation method to segment multimodal MRI data, which combined the advantages of 2D and 3D CNNs to capture spatial context features. The results showed that the DSC of the tumor, tumor core, and tumor enhancement region were 0.918, 0.883, and 0.854, respectively, which outperformed the accuracy of the segmentation algorithm.

#### 4.4.2 Application in glioma classification

DL can help physicians identify whether a medical image is a type of classification problem in the ML field. The pathological type of brain tumors and the classification of brain gliomas largely determine the treatment plan and overall prognosis; therefore, accurate classification of brain tumors is of great clinical significance. In recent years, many researchers have fully explored the deep characteristics of the MRI dataset using CNN and achieved the classification of gliomas, which shows the feasibility of this approach and makes a difference in the trend of precision medicine. There are several applications for the classification of gliomas.

DL can be used for the detection of gliomas, which is used to classify images containing gliomas using a database of MRI images. The output was an MRI image marked as normal or abnormal. Tumor detection is generally the first step in clinical diagnosis and treatment and can be applied to disease screening and routine physical examinations on a large scale. Abd‐Ellah et al.^80^ used a combination of AlexNet and an error‐correcting output code support vector machine (ECOC‐SVM) to evaluate cranial images and obtained a 99.55% detection accuracy in the Response(Rider)Neuro MRI database.

Furthermore, DL can be used to identify different brain tumors, including gliomas. Paul et al.^81^ used a contrast‐enhanced T1‐weighted imaging (CE‐T1WI) MRI dataset based on the CNN method to classify gliomas, meningiomas, and pituitary tumors. The classification accuracy was 90.26%. Subsequently, Swati et al.^82^ used a pretrained CNN model and proposed a transfer learning‐based strategy for classification with an accuracy of up to 94.82%. Deepak and Ameer^83^ further combined a CNN with a SVM, and the accuracy was improved to 97.8%.

In addition to the qualitative diagnosis of brain tumors, DL has also achieved a degree of glioma prediction. Yang et al.^84^ used GoogLeNet and AlexNet models for LGG and HGG classification, and the classification accuracies were 94.5% and 93.8%, respectively, indicating that GoogLeNet had better classification performance. Ge et al.^85^ incorporated multimodal MRI into a multi‐stream 2D CNN model to achieve the classification of LGG and HGG, of which CE‐T1WI had the highest performance, with an accuracy rate of 83.87%. By the fusion of multimodal features, the accuracy improved by approximately 7%. Mzoughi et al.^86^ proposed a multiscale 3D CNN architecture to classify LGG and HGG based on multimodal MRI datasets, and used data augmentation technology to preprocess the images, and its classification accuracy increased from 82.5% to 96.4%, outperforming the 2D CNN model. Diffusion tensor imaging (DTI) is a new method for describing brain structure and is a special form of MRI. Based on the CNN model, Zhang Z et al.^87^ extracted deep features from a DTI dataset for glioma classification. The results showed that the accuracy of distinguishing LGG from HGG was 94%, and the accuracy of distinguishing WHO III from WHO IV gliomas was 98%, indicating that the deep features extracted from brain DTI images helped to distinguish different levels of gliomas.

DL also has important applications in identifying postoperative glioma recurrence and treatment‐related effects (TRE). For example, Bacchi et al.^88^ used CNNs based on a combination of DWI + FLAIR sequences and achieved a model accuracy of 82%. Tang F et al.^89^ developed a fully automatic DL method based on multisequence MRI guidance. The deep feature fusion model is a multi‐sequence MRI‐guided CNN model that iteratively learns CT images and multiple sequences simultaneously. These two features were then combined to generate the classification results.

The results of all this research show that DL is helpful for the classification of gliomas. However, single‐center research is still a main problem and limitation in these experiments, and the problem of small sample size also requires further multicenter research and large samples to verify their feasibility.

#### 4.4.3 Application in mutation prediction

In the trend of precision medicine, gene mutation status has been added to glioma subtypes to create more personalized treatment plans for patients. Knowing that tumor gene status, such as IDH, O^6^‐methylguanine‐DNA methyltransferase (MGMT), and 1p/19q, are beneficial for treatment planning and prognosis prediction,^90^, ^91^ more studies have explored the value of DL in predicting glioma gene types in recent years. Different molecular subtypes of gliomas differ in tumor susceptibility location and therapeutic sensitivity, leading to different extrinsic phenotypes, such as tissue and cellular features, including shape, edge, location, and texture. These features or other abstract features may be learned and acquired by DL models for classification and prediction. Because genotyping by pathological tissue detection after surgery takes a long time, which delays the treatment of glioma patients, there is an urgent need to predict glioma genotypes based on MRI features before surgery to meet the needs of clinical treatment.

DL networks can be used to predict gene mutations. One of the commonly used types of DL networks for this task is CNNs. The input data type for CNNs is usually the gene expression data, which is a collection of measurements of the activity of genes in a cell, tissue, or organism, often obtained through RNA sequencing experiments. To train a CNN for gene mutation prediction, the data is first preprocessed, normalized, and cleaned to ensure data quality. The data is then split into training and testing datasets. The training data is used to train the CNN model to learn the patterns and relationships between gene expression data and the presence or absence of a particular mutation. During training, the CNN model adjusts its weights and biases based on the input data and the known output (mutation status). The model continues to train until the loss function reaches a minimum, indicating that the model has learned to predict the mutation status accurately.

IDH1 mutations are closely associated with glioma patients' survival. Li Z et al.^92^ modified a CNN model to have six convolutional layers and a fully connected layer with 4096 neurons, which was used to segment tumors and predict the IDH1 mutation status in LGG patients, by validating models on a dataset of 151 patients with LGG and comparing performances with normal radiomics approaches, results showed that the AUC of the normal radiomics methods and DL methods was 0.86 and 0.92, respectively, for IDH1 estimation. Furthermore, paired *t*‐tests and *F*‐scores were used to select CNN features that identify IDH1 and proved DL's ability of extracting deep information from medical images.

MGMT contributes to DNA repair, and methylated MGMT inhibits DNA repair, resulting in resistance to chemotherapeutic drugs. Korfiatis et al.^93^ used three ResNet models to predict the methylation status of the MGMT promoter, and showed that the ResNet50 model outperformed the ResNet34 and ResNet18 models, with accuracies of 94.90%, 80.72%, and 75.75%, respectively. Yogananda et al.^94^ used 247 subjects' brain MR imaging and corresponding genomic information obtained from TCIA and TCGA where 163 subjects had a methylated MGMT promoter as input, a T2WI‐only network (MGMT‐net) was developed and trained using 3D‐dense‐UNets to determine MGMT promoter methylation status and simultaneous single‐label tumor segmentation. Results showed that the DL based method surpasses traditional histologic and molecular methods.

The detection of the 1p/19q co‐deletion is of great significance in diagnosing oligodendroglioma and determining the prognosis of patients. Compared with patients with 1p/19q non‐deletion, patients with IDH mutation combined with 1p/19q co‐deletion had a better prognosis. The imaging manifestations of gliomas with combined deletion of chromosome 1p/19q have certain characteristics that enable DL to predict its status through MRI images. Akkus et al.^95^ used 159 LGG with three image slices each who had biopsy‐proven 1p/19q status and preoperative postcontrast‐T1 (T1C) and T2 images as input, successfully predicted the 1p/19q co‐deletion status in LGG patients based on a multiscale CNN model, with an accuracy of 87.70% and sensitivity of 93.3%. The CNN model trained by Chang et al.^96^ achieved multigene prediction of glioma, including IDH1 mutation, MGMT methylation, and 1p/19q co‐deletion status, with an accuracy of 94%, 83%, and 92%, respectively, suggesting that the status of multiple genes can be simultaneously predicted using CNN. High accuracy has been reported in these studies, but several limitations still exist including generally small datasets, a lack of studies with multiple centers training datasets and independent testing datasets, and a lack of studies predicting IDH and 1p/19q together. While DL combining with gliomas MRI shows great potential as a noninvasive approach for glioma genotyping, these limitations need to be addressed before it truly makes clinical translation.^97^

#### 4.4.4 Application in survival prediction

With the development of medical technology, the treatment of brain glioma has made great progress, but the survival rate of patients with high‐grade glioma is still very low; in particular, the median overall survival of glioblastoma patients is only about 12–15 months. Therefore, there is an urgent need to fairly predict glioma progression‐free survival and overall survival. Previous studies on survival prediction have included patient age, sex, physical status, extent of resection, tumor type, tumor site, and tumor size as prognostic factors; however, these indicators have limitations that cannot reflect intratumoral heterogeneity, which can also influence patients' conditions. MRI‐based radiomics has been proven to be useful for predicting the survival of gliomas without considering glioma grade.^98^ However, manually selected radiomic features are subjective and sensitive to changes in the observation environment. DL has the ability to identify the deep and abstract features in tumors that the naked eye cannot capture, and its automated workflow can avoid subjectivity influences, which makes it more helpful to predict patient survival. DL can also be integrated with MRI radiomics to predict survival and tumor‐infiltrating macrophages in gliomas.^99^

Nie et al.^100^ employed DL to automatically extract deep features from multimodal and multichannel MRI datasets to predict overall survival in patients with HGG. These deep features, along with manually selected clinical features such as age, sex, and health condition, were then fed into the SVM to predict overall survival. This DL‐SVM approach achieved an accuracy of 90.66%. Considering the close relationship between genotypes such as IDH, MGMT, and 1p19q and patient survival, Tang Z et al.^101^ proposed a multitask CNN model to jointly complete tumor genotype and overall survival prediction tasks. The results of this method showed that features associated with tumor genotype significantly improved the accuracy of predicting overall survival, and features associated with overall survival also improved the accuracy of predicting genotype, which in turn proved the correlation between genotype and survival. These studies also contribute significantly to progress in precision medicine.

## 5 LIMITATIONS AND PROSPECTS

We are living in an era of digital information explosion. Medical information, too, has gradually become predominately digital and easy to preserve. DL, however, often has higher requirements for training data, not only in terms of quantity but also quality. Marking medical images require professional doctors, and thus good marked medical images are difficult to obtain. Additionally, there will be noise in the data, even for professional doctors, and the results may also vary per doctor. Owing to the particularity of medical information and privacy‐protection policies, well‐processed datasets that doctors can obtain are limited. Unwell‐trained models are easy to overfit and have poor generalization, which cannot be applied to medicine. Incomplete sample coverage may lead to model performance dropping due to the center effect; for example, AlBadawy et al.^102^ investigated the segmentation performance of CNN on glioblastoma MRI data from two institutions. The results indicate that the segmentation accuracy (DSC was 0.68 ± 0.19) using data from different institutions was significantly lower than that using data from the same institution Precision (DSC 0.72 ± 0.17). Thus, data from different institutions vary in imaging equipment, image acquisition parameters, and contrast agent use, which may affect the image quality and segmentation accuracy.

Owing to a lack of training samples, learning approaches using small samples can alleviate this problem to a certain extent. The first proposal is using transfer learning; this is performed by moving the trained model on a task (source domain) to another task (target domain),^103^, ^104^, ^105^ because the source domain has a large number of training samples. After large‐scale pretraining on the source domain, the DL model only needs to be fine‐tuned in the target domain with few samples. For example, Med3D can be pretrained on a large number of heterogeneous public datasets, which is a shared encoder segmentation network, and then transferred to other tasks such as lung segmentation.^106^ This method can significantly improve segmentation and classification accuracy in the absence of training data.

For the gap (domain shift) problem between the target domain and the source domain, Chen J et al. also proposed a novel unsupervised domain adaptation method^107^ to alleviate the performance degradation caused by domain shift.^108^, ^109^ The method only requires the data and annotations of the source domain and some images of the target domain and can realize the adaptation of the two domains without the annotation of the target domain. Additionally, such a domain‐adaptive boosting algorithm can improve generalization across centers.^110^ Furthermore, to address the lack of large datasets, GANs have been used to produce large datasets of high‐resolution images using image reconstruction technology,^111^, ^112^, ^113^, ^114^ which could help increase the number of DL applications in the medical field and drive precision medicine in the future.

DL has already made significant strides in the clinical stage of glioma, particularly in the areas of diagnosis, prognosis, and treatment planning. However, there is still much work to be done before it can be considered fully integrated into clinical practice.

One of the main challenges is the need for large, diverse datasets to train and validate DL models. Glioma is a complex and heterogeneous disease, and obtaining high‐quality data that accurately reflects this complexity can be difficult. Additionally, there are ethical and regulatory considerations that must be addressed when using patient data for research. Another challenge is the need for interpretability and transparency in DL models. Clinicians need to be able to understand how the models are making predictions and be confident in their accuracy before incorporating them into clinical decision‐making. Despite these challenges, there have been promising developments in the field of DL and glioma. Researchers have developed DL models that can accurately predict patient outcomes and guide treatment planning based on imaging data. The potential benefits of DL in the clinical stage of glioma make it an exciting area of research and development.

The field of DL in clinical and glioblastoma research is poised to undergo significant growth and advancement in the near future. This growth will be largely driven by the increasing availability of medical data, which is making it possible to analyze and understand complex disease processes in ways that were previously not possible. In addition, the development of more sophisticated algorithms is providing researchers with powerful new tools for analyzing and interpreting this data.

Furthermore, the integration of multiple types of data and information is also contributing to the future development of DL in these areas. By combining data from sources such as imaging studies, genomics, and clinical records, researchers can gain a more complete understanding of disease processes and develop more effective treatments. As medical data continues to become more widely available, and as algorithms and data integration techniques continue to improve, it can be expected to see significant progress in ability to diagnose and treat diseases like glioblastoma, ultimately leading to better outcomes for patients.

## AUTHOR CONTRIBUTIONS

**YIHAO LIU:** Conceptualization (equal); writing – original draft (equal). **Minghua Wu:** Funding acquisition (equal); project administration (equal); writing – review and editing (equal).

## FUNDING INFORMATION

This work is supported by the National Natural Science Foundation of China (82073096) and Key Research and Development Plan of Hunan Province (2020SK2053).

## CONFLICT OF INTEREST STATEMENT

The authors declare no conflicts of interest.

### PEER REVIEW

The peer review history for this article is available at https://www.webofscience.com/api/gateway/wos/peer-review/10.1002/btm2.10553.

## TRANSLATIONAL IMPACT STATEMENT

This essay explores the application of deep learning in precision medicine, with a focus on glioma. By analyzing medical data and extracting multilevel features, deep learning methods can help doctors assess diseases automatically and monitor patients' health. However, more research is needed on multi‐omics data to provide more comprehensive and accurate diagnosis in precision medicine and glioma.

## Data Availability

Not applicable.
